# Effects of Curing Defects in Adhesive Layers on Carbon Fiber–Quartz Fiber Bonded Joint Performance

**DOI:** 10.3390/polym16101406

**Published:** 2024-05-15

**Authors:** Xiaobo Yang, Miaomiao Zhang, Lihua Zhan, Bolin Ma, Xintong Wu, Cong Liu, He Xiang

**Affiliations:** 1 College of Mechanical and Electrical Engineering, Lanzhou Jiaotong University, Lanzhou 730070, China; zhangmmiao11@163.com; 2 Institute of Light Alloy, Central South University, Changsha 410083, China; mabolin_csu@163.com; 3 School of Advanced Manufacturing, Nanchang University, Nanchang 330029, China; wuxintong@ncu.edu.cn; 4 College of Mechanical and Electrical Engineering, Central South University, Changsha 410083, China; 15842820170@163.com; 5 Ningbo Branch of Ordnance Science Institute of China, Ningbo 315103, China; xianghe68@126.com

**Keywords:** carbon fiber–quartz fiber bonded joint, films’ cured thermophysical parameters, prefabricated defects, different void defects, mechanical properties

## Abstract

Due to their mechanical load-bearing and functional wave transmission, adhesively bonded joints of carbon fiber–quartz fiber composites have been widely used in the new generation of stealth aviation equipment. However, the curing defects, caused by deviations between the process environment and the setting parameters, directly affect the service performance of the joint during the curing cycle. Therefore, the thermophysical parameter evolution of adhesive films was analyzed via dynamic DSC (differential scanning calorimeter), isothermal DSC and TGA (thermal gravimetric analyzer) tests. The various prefabricating defects within the adhesive layer were used to systematically simulate the impacts of void defects on the tensile properties, and orthogonal tests were designed to clarify the effects of the curing process parameters on the joints’ bonding performance. The results demonstrate that the J-116 B adhesive film starts to cure at a temperature of 160 °C and gradually forms a three-dimensional mesh-bearing structure. Furthermore, a bonding interface between the J-116 B adhesive film and the components to be connected is generated. When the curing temperature exceeds 200 °C, both the adhesive film and the resin matrix thermally degrade the molecular structure. The adhesive strength weakens with an increasing defect area ratio and number, remaining more sensitive to triangle, edge and penetration defects. By affecting the molecular structure of the adhesive film, the curing temperature has a significant impact on the bonding properties; when the curing degree is ensured, the curing pressure directly impacts the adhesive’s performance by influencing the morphology, number and distribution of voids. Conversely, the heating rate and heat preservation time have minimal effects on the bonding performance.

## 1. Introduction

To improve survivability in a complex battlefield environment, higher requirements are put forward for the maneuverability and stealth of new-generation military aviation equipment. The use of advanced composites is an effective way of meeting the dual structural and functional needs of military aviation equipment [1,2,3]. Due to their high specific strength and modulus, carbon fiber composites are applied in complex load-bearing conditions, while, under the premise of satisfying the service conditions, quartz fiber composites can achieve wave transmission in a wide frequency range [4,5,6]. Compared with traditional metals and wave-transmitting coating materials, carbon fiber–quartz fiber composite components can achieve a service performance enhancement of 20% to 40%, an improvement of 30% to 50% in wave-transparent properties and a comprehensive cost reduction of more than 25%. They have become an indispensable part of the new generation of stealth fighters. Currently, advanced composites have become one of the most important aviation and aerospace structural materials after aluminum alloys, titanium alloys and steels, and they are widely used in aircraft structures, ranging from non-bearing and sub-bearing structures to main bearing structures [7,8]. The proportion of the usage and the used parts have been taken as the key indexes to measure the advancement of the aircrafts and international competitiveness. Aiming for the multifunctional composite components that cannot be cured integrally, the bonding process of assembly components has been widely focused on by virtue of its operational feasibility, which can avoid the problems of excessive stress, weakened stealth and increased structural weight caused by mechanical connection [9,10,11]. Carbon fiber–quartz fiber composite bonded joints are mainly cured using an autoclave [12,13]. However, when the autoclave’s curing temperature and pressure parameters deviate from those based on the adhesive film setting, void and delamination defects will be generated, which weaken the mechanical service performance [14,15,16]. Thus, the effects of shape, location and area ratio of defects within the adhesive layer on the bonded joints performance should be studied. Based on this, association rules regarding curing process parameters, defect characteristics and mechanical properties are revealed. The curing process requirements of carbon fiber–quartz fiber bonded joints with fewer defects and a higher performance can be clarified, which further promote the application of advanced composite materials in new-generation aviation equipment [4,17].

In recent years, with the sustainable development of the adhesive film system and bonding process, research teams have studied the influence of defect causes and types in adhesive layers on joint performance. Kumar et al. [18] used ethylene tetrafluoroethylene (ETFE) to prefabricate the defects and considered three defect sizes of 25%, 48% and 70% of the total bonding area, respectively. The failure loads of the specimens with and without defects were compared. They found that the failure load decreased with the defect size, especially for the specimen with a defect size of 70% of the bonding area, for which the failure load decreased by 65%. Fame et al. [19,20] studied the effect of shape, size and location of defects on joint performance using Teflon strips to prefabricate defects, and it was found that triangular defects weakened the performance more obviously compared with rectangular, square, circular and elliptical defects when the defect area ratio was fixed; the defects located at the free end showed an outstanding decrease. Liu et al. [21] conducted a visual investigation of the damage evolution of wide-bond lap joints with semicircular process-induced defects. The results indicated that the cracks gradually expanded in two directions but mainly developed along the transverse direction (i.e., perpendicular to the loading direction) until the joint width was reached. Simultaneously, some scholars have carried out corresponding curing process research with the goal of achieving a high bonding strength. For example, Yang et al. [22] investigated the effects of lap length, adhesive thickness and ply type on the maximum peel and shear stresses of T700/Epoxy composite single-lap joints. It was found that the adhesive thickness and ply type primarily affected the peel lap length of the joints, and that an increased lap length could improve the bond strength within a certain range. Elhannani et al. [23] have analyzed the effect of different sizes, shapes and locations of defects, overlap lengths and stress conditions on the shear stress in the adhesive layer by the finite element method. It was found that the point of maximum stress is always located at the edge of the glued joint region regardless of the size of the defect. When the lap length is important, the defects have less effect on the strength of the joint. Moreover, previous studies [24,25,26] on composite surface treatment methods include examinations of mechanical treatment, peel-layer technology and laser treatment, focusing on optimizing the process parameters to improve the composite surfaces in order to enhance the bond strength and fracture toughness of the bonded joints. However, few studies have comprehensively investigated and analyzed the effect of the area ratio, location, morphology and penetration of the defects as relevant variables on the model of joint failure. In addition, the effect of the adhesive film’s thermal physical parameters on the bonding performance was fragmented. At the same time, the processes of existing studies focus more on physically or chemically modifying the bonding surface to improve the adhesive bonding strength. However, regarding low-defect manufacturing, there are relatively few studies on the optimization of the adhesive process. Aiming to address the above problems, in this study, we performed tests on adhesive films’ thermophysical parameters in order to obtain the dynamic DSC, isothermal DSC and TGA characteristics of J-116B film. Secondly, looking at pre-embedded polytetrafluoroethylene (PTFE) defects, the influence of defect area ratio, location, morphology and penetration on the bonding strength were systematically studied, and a correlation law between the defect form and bonding performance was constructed. Finally, orthogonal experiments, considering the pressure, heating rate, temperature and heat preservation time, were designed to determine the key process factors that affect the bonding strength.

## 2. Experimental Section

### 2.1. Materials

In this study, the unidirectional carbon-fiber-reinforced bimaleimide resin prepreg ZT7H/5429 with a single-layer thickness of 0.125 mm and the woven quartz-fiber-reinforced bimaleimide prepreg QW280/5429 with a single-layer thickness of 0.250 mm were supported by AVIC Composites Co., Ltd., Shenzhen, China. Due to the single-layer thicknesses of the two prepregs being different, the prepreg ZT7H/5429 was stacked using lay-ups of [45/90/−45/0/45/90/−45/0/45/90]s to produce carbon fiber composite laminates and quartz composite laminates, which were fabricated by stacking prepreg QW280/5429 in the order of [45/90/−45/0/45]s. The thicknesses of different laminates are 2.5 mm. Meanwhile, the anisotropy of the laminates could be avoided by using the above stacking method, which ensures the mechanical properties of the plate.

**Figure 1:**
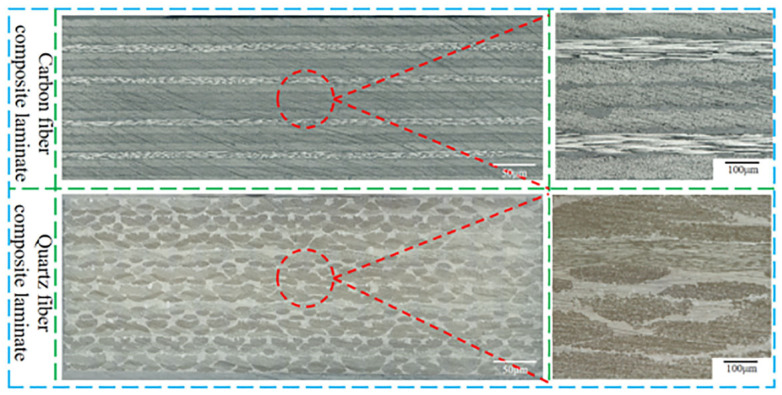
The micrographs of carbon fiber composite laminate and quartz fiber composite laminate.

Carbon fiber composite laminates and quartz composite laminates should have no internal void defects to clarify the effect of adhesive layer defects on the bonded joint performance in Figure 1. J-116B is an epoxy adhesive film supplied by Kuang-Chi Technologies Co., Ltd., Shenzhen, China. The single-layer thickness is 0.2 mm. PTFE was pre-embedded into J-116B as prefabricated defects.

### 2.2. Testing Thermal Physical Parameters of Film

The dynamic DSC and isothermal DSC experiments of the film were carried out by using the DSC214 differential scanning calorimeter produced by NETZSCH, and the sample weight is 15 ± 0.5 mg. During the whole experiment, nitrogen was used as the protective gas. In the dynamic DSC experiment, the heat release of films was measured when the samples were separately heated at constant rates of 3 °C/min, 5 °C/min, 7 °C/min and 10 °C/min, and the temperature rise range was 25–300 °C. The samples were, respectively, heated from 25 °C to 140 °C, 160 °C, 180 °C and 200 °C at the rate of 3 °C/min for 400 min in the isothermal DSC experiment. The curing heat release of resin defined as $∆H_{t}$ at any time can be obtained by integrating the relationship curve between the exothermic rate dH/dt and temperature T in the curing reaction stage measured by DSC. The curing degree at any time can be gained according to Equation (1). Moreover, a thermogravimetric analyzer system was used for the TGA experiment of film and 5429 resin matrix, with a sample weight of 20 ± 0.2 mg. Film samples and resin samples were separately heated from 30 °C to 800 °C at the rate of 10 °C/min under the argon atmosphere with a flow rate of 50 mL/min until the end.

(1) $$\alpha (T)=\frac{∆H_{t}}{∆H_{0}}$$

where $∆H_{t}$ (J/g) is the heat release of the resin curing to time *t*, and $∆H_{0}$ (J/g) is the total heat release of the resin curing reaction.

### 2.3. Adhesive Bonded Specimen and Defect Design

**Figure 2:**
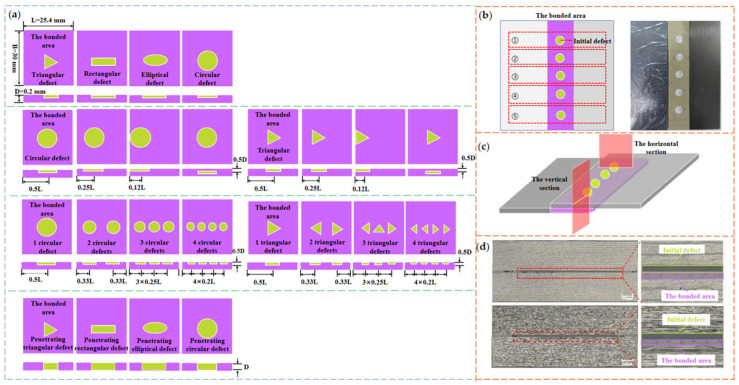
Fabrication of artificial defect samples. (**a**) Defect configurations in the bonded area, (**b**) typical artificial defect setup, (**c**) schematic of sampling direction, (**d**) micrographs of artificial defect in different directions.

The manufacturing dimensions of the carbon fiber and quartz fiber composite laminates were 75 mm × 160 mm (width × length), the bonded area size was 30 mm × 160 mm (width × length) and the film thickness was 0.2 mm. The mechanical test specimens for five groups with the same defect type can be taken out along the length of the laminate, and the subsequent area ratio of prefabricated defects is the ratio of the defect area to the adhesive area in the individual specimen. Combined with the defect prefabrication method in reference [27], the morphologies of the prefabricated defects were designed as triangle, rectangle, ellipse and circle through the pre-embedded PTFE [28,29]. Despite the fact that the formation mechanism of PTFE defects is different from that of actual void defects, the effect of the defect parameters, such as morphology, number and location, is similar on the failure mechanism of joints. With reference to the void content requirements for aviation aerospace components, the area ratios of the four types of defect were selected at 1% (the void content standard for major bearing aviation components), 5% (the key void content for a significant reduction in the performance of aerospace load-bearing components) and 10% in order to analyze the impact of the defective area ratio on the strength of the bonded joints. As shown in Figure 2a, the defect morphologies were selected as triangular and circular with an area ratio of 5%, and the distances between the geometric center of defects and the edge were, respectively, considered at 0.12 L (at edge), 0.25 L and 0.5 L in order to examine the effect of defect locations on the bonding strength. As a control, defects were introduced in the thickness direction with a geometric center distance of 0.5 D from the adhesive layer’s surface.

In order to analyze the impact of different defect numbers on the bonding strength, the number of triangular and circular defects was set from 1 to 4. The total area of defects with different numbers accounted for 5%. The defects in the above experiments were all non-penetrating and the thickness was 0.1 mm, which was half the thickness of the adhesive layer. The thickness of the penetrating defect was equal to that of the adhesive layer. The morphologies of the penetrating defects were triangular, rectangular, elliptical and circular, with an area ratio of 5%. Figure 2b,c demonstrate that a verification test was conducted to ascertain the defects that could remain at the designated location during the curing process. The defect size and spacing distance in the horizontal and vertical directions were observed under a metallographic microscope. The results showed that the error of prefabricated defect size and location could be controlled within 4% and 8%, respectively.

### 2.4. Orthogonal Experimental Method of Bonding Process

In the bonding cycle process, the process parameters directly affect the number, morphology and distribution of curing defects in the bonded area, thus affecting the mechanical quality [15,18,20,30]. In order to study the above relationship, curing pressure, heating rate, heat preservation temperature and heat preservation time were selected to carry out orthogonal experiments [31,32]. The level of each factor was set to 4 to form the L16 (4^4^) curing experiments, simulating the deviation of the curing parameters from the setting parameters, as shown in Table 1. The bonded joints were cured under each group of process parameters to generate defects for different types.

#### 2.4.1. Difference Analysis Method

In the orthogonal tests, sixteen composite laminates were fabricated by different curing parameters, and their interlaminar shear strengths (ILSSs) were used for the difference analysis.

The sum of the ILSS results can be expressed as follows:

(2) $$T_{ij}=\sum_{k=1}^{m}y_{ij}^{k},$$

where $y_{ij}$ is defined as the strength result of i level in j factor, and m is the number of factors.

The mean value of the sum of ILSS results can be described as follows:

(3) $$M_{ij}=\frac{l}{n}T_{ij},$$

where n is the total number of orthogonal experiments, and l is the number of levels.

The difference value of the j factor is expressed by $R_{j}$, and the corresponding mathematical model is as follows:

(4) $$R_{j}=maxM_{ij}-minM_{ij},$$

The size of the $R_{j}$ value reflects the degree of influence of various factors on the test results.

#### 2.4.2. Variance Analysis Method

The variance analysis can be used to obtain the influence weight of each factor on bonding strength through the F-test. The square sum of total deviations ${SS}_{T}$ of ILSS results obtained by orthogonal experiments can be expressed by the following equation:

(5) $${SS}_{T}=\sum_{j=1}^{n}{\left(x_{i}-\bar{x}\right)}^{2},$$

where $x_{i}$ is the mean value of ILSSs for the i group, and $\bar{x}$ is the mean value of all experimental results.

Regarding $L=\sum_{i=1}^{n}\left.x_{i}\right.$, the square sum of the *j* factor deviation can be expressed by the following equation:

(6) $${SS}_{j}=\sum_{i=1}^{l}{\left({\bar{L}}_{ij}-\bar{x}\right)}^{2},$$

Additionally, the difference between ${SS}_{T}$ and ${SS}_{j}$ is indicated by the square sum of deviations ${SS}_{e}$, which is caused by random errors. The equation of ${SS}_{e}$ can be expressed as follows:

(7) $${SS}_{e}={SS}_{T}-\sum_{j=1}^{m}{SS}_{j},$$

Finally, the value of *F* is calculated to test the influence of the *j* factor on the results, and when the *F* is higher than the critical value, the factor has a significant impact on the results. The calculation equation of *F* is as follows:

(8) $$F=\frac{{SS}_{j}(N-n)}{SS_{e}\left(n-1\right)},$$

where N is the total number of samples in the j factor, and N=n × l.

### 2.5. Mechanical Performance Testing

**Figure 3:**
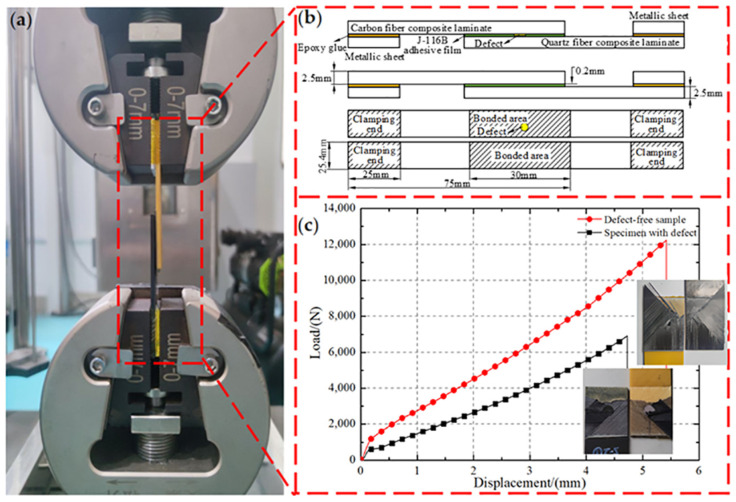
The lap shear test of bonded joint. (**a**) The test set up of the bonded joint in tension, (**b**) the schematic of lap shear sample, (**c**) the representative load–displacement curve of lap shear test.

Compared with other mechanical tests, the single-lap experiment could well reflect the mechanical properties of the bonded area. The geometric model of the tensile specimen, which includes a typical type of prefabricated defects and non-defects, is depicted in Figure 3a,b. According to the American standard ASTM D3165 [33], the dimensions of the test sample were 120 mm × 25.4 mm (length × width), and the dimensions of the bonded area were 30 mm × 25.4 mm (length × width). Five samples were taken from each group, and both ends of the sample were fixed by metal gaskets using epoxy adhesive to increase friction and avoid the sample disengaging from the fixture. Tensile tests were performed using the ASTM testing machine, and the loading rate was controlled by a constant displacement rate of 1 mm/min throughout the tensile process. Meanwhile, the load and beam displacement were recorded at 10 Hz until the bonded area peeled off under loading. The force–displacement curves are shown in Figure 3c. The load first increased and then irreversibly decreased, which is similar to the evolution force law in refs. [27,32]. Compared with the analysis of multiple force–displacement curves, the ILSS analysis is utilized to better reflect the effect of defects in adhesive film and process parameters on joint performance. The bonding strength of the specimens was determined according to Equation (9).

(9) $$\sigma =\frac{F_{max}}{bl}$$

where *F*_max_ is the maximum tensile force, *b* is the width of samples and *l* is the lap length.

## 3. Results and Discussion

### 3.1. Thermophysical Parameter Analysis of Film

#### 3.1.1. Non-Isothermal and Isothermal Parameter Analysis of Film

**Figure 4:**
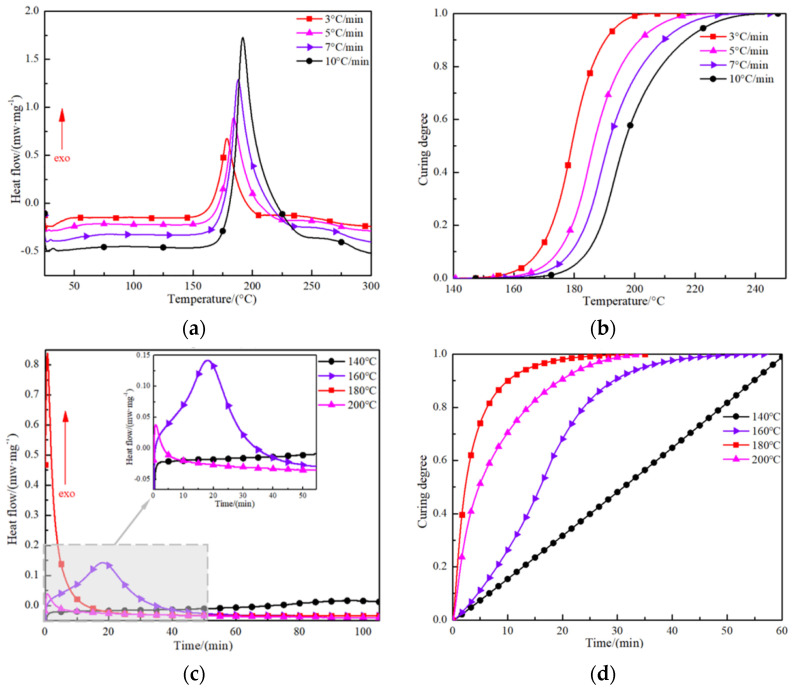
The non-isothermal and isothermal scanning DSC tests. (**a**) DSC curves for adhesive film as received treated at various heating rates, (**b**) degree of cure for various heating rates, (**c**) DSC curves for adhesive film as received treated at various isothermal temperatures, (**d**) degree of cure evolution at various isothermal temperatures.

The curing exothermic curves of J-116B film at different heating rates are presented in Figure 4. At any heating rate, there is a single exothermic peak that indicates only one curing reaction within 300 °C. In the initial stage of heating, the carbon–oxygen bonds in the epoxy groups are broken, and the nitrogen–hydrogen bonds in the primary amino groups in the coupling agent are disconnected, resulting in the adhesive film being in the heat absorbing stage. When the temperature reaches 160 °C, a crosslinking reaction inside the film gradually occurs, developing a three-dimensional network structure and releasing heat [13,34,35] (see Figure 4a). With increasing heating rate, the exothermic peak gradually shifts to the right, and the curing exothermic reaction of the film becomes more drastic, leading to a reduction in the time to complete a curing process (see Figure 4b). However, it should be noted that, as the heating rate increased, the heat released from the curing reaction of films led to an increase in the temperature difference in the exothermic process; this easily aggravated the uneven distribution of the temperature field generated during the curing process.

At the same time, the curing degree of the thermosetting adhesive film increased with time in a constant temperature environment. With the decrease in the heat preservation temperature, the time to reach the curing exothermic peak lengthened, and there was no obvious exothermic peak at 140 °C, indicating that the curing reaction progressed slowly at this heat preservation temperature, as shown in Figure 4c. Figure 4d illustrates that the curing time increases non-linearly with the increase in heat preservation temperature. The heat preservation time for the curing degree to reach 0.95 at 160 °C was 35 min, while the curing degree reached 0.95 at 180 °C and 200 °C for 15 min and 25 min, respectively. The reason for the above results is that the film’s exothermic peak temperature was relatively low at a lower heating rate. In this moment, a heat preservation temperature of 180 °C was more conducive to stimulating the film curing reaction and promoting the three-dimensional network structure to form quickly [36]. There is further indication that the temperature range of the curing exothermic peak needs time to activate the curing reaction and maintain polymer bond formation.

#### 3.1.2. TGA Test for Film and Resin

**Figure 5:**
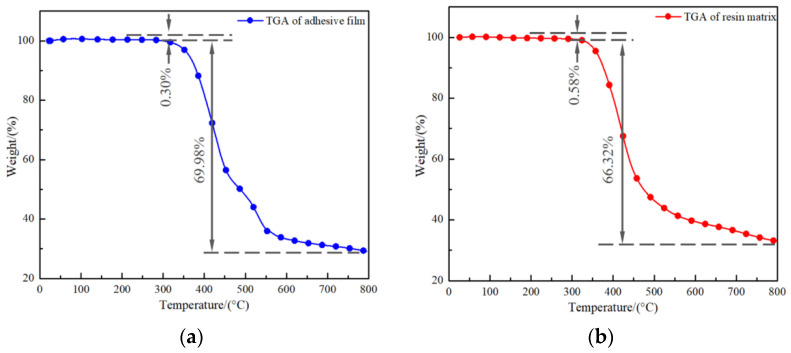
The typical TGA response of adhesive film and resin samples. (**a**) The TGA curve for adhesive film sample, (**b**) the TGA curve for resin sample.

Figure 5 provides the TGA curves for the film and resin; all test samples showed two loss stages. In the first stage at about 200 °C, the film and resin loss was 0.30% and 0.58%, respectively, probably caused by the volatilization of small molecular organic matter [21,37], as shown by the slow decline characteristics in the early curve. In the second stage, the film and resin are at around 300 °C; then, the samples’ mass suddenly and rapidly reduced with the increased temperature to achieve 69.98% and 66.32%. The most important reason for this is the complete decomposition of the adhesive film and resin during the heating process [38,39]. Therefore, when the setting temperature of the carbon fiber–quartz fiber laminate bonded joint does not exceed 200 °C, the mechanical properties of the cured bonded joint are not affected by thermal decomposition.

### 3.2. Effect of Bonding Defects on Mechanical Performance

#### 3.2.1. Effect of Defect Shape and Size

Aiming for the types of defects shown in the first line of Figure 2a and setting their area ratios at 1%, 5% and 10%, respectively, the effects of defect shapes and sizes on the performance of joints shown in Table 2 can be obtained. The relative failure load of the specimen is defined as the percentage value of the adhesive joint strength with defects and non-defects. The value of the latter is 16.33 MPa and the relative failure strength is 100%.

When the defect area ratio is 1%, the bonding strength with triangle, rectangle, ellipse and circle defects decreases by 8.39%, 6.80%, 4.65% and 3.80% when compared with those with no defects. As the area ratio increases to 5%, the bonding strength with different shape defects decreases by 12.74%, 10.41%, 9.31% and 6.98%, respectively. At a ratio of 10%, the strength decline is 13.47%, 12.31%, 10.04% and 7.16%, respectively. Under different area ratios, the impact of defect shape on the adhesive shear strength is ranked as follows: circle less than ellipse, ellipse less than rectangle and rectangle less than triangle. The bonding strength gradually decreases with the sharpening of the defect’s geometric angle. This is caused by the fact that the stress concentration in the bonding zone is due to a sharp angle appearing in the process of defect morphology evolution; tip-splitting [30] easily occurs during the energy dissipation process, which leads to the initiation and propagation of cracks [32,40,41]. Meanwhile, examining various morphological defects, it can be seen that the defect has a small effect on the bonding strength at an area ratio of 1%, and the strength reduction is kept below 8.5%. As the defect size enlarges to the range from 5% to 10%, the bonding strength declines rapidly. Furthermore, the larger the defect area ratio, the more sensitive the bond strength is to the defect shape.

**Figure 6:**
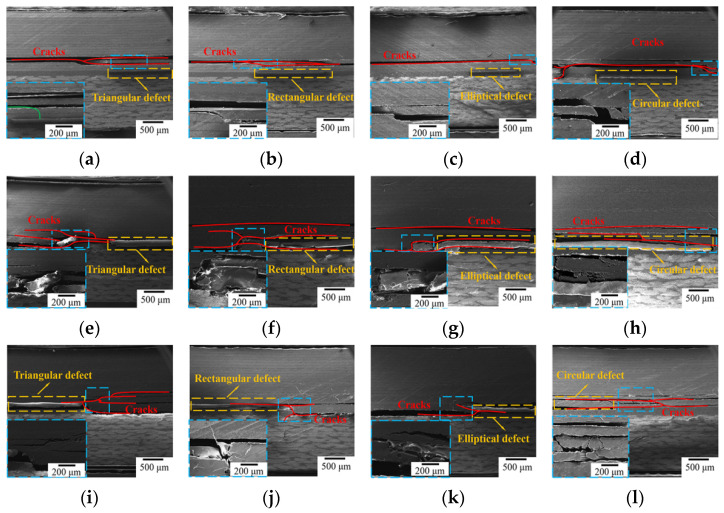
Scanning electron micrographs of samples having defects with different shapes and area ratios. (**a**) Triangular defect with 1% area ratio, (**b**) rectangular defect with 1% area ratio, (**c**) elliptical defect with 1% area ratio, (**d**) circular defect with 1% area ratio, (**e**) triangular defect with 5% area ratio, (**f**) rectangular defect with 5% area ratio, (**g**) elliptical defect with 5% area ratio, (**h**) circular defect with 5% area ratio, (**i**) triangular defect with 10% area ratio, (**j**) rectangular defect with 10% area ratio, (**k**) elliptical defect with 10% area ratio, (**l**) circular defect with 10% area ratio.

SEM damage maps of bonded joints containing defects with different shapes and area ratios under tensile loading are shown in Figure 6. With an area ratio of 1%, the prefabricated defects are not the key factor inducing the initiation and propagation of cracks. Based on the results depicted in Figure 6a–d, the stress is concentrated at the end of bonding joint between the carbon fiber and quartz fiber laminates; the cracks mainly extend along the interface between the third layer (−45°) and the fourth layer (0°) of the carbon fiber laminate. These observations suggest that the adhesive bonding ability between the adhesive film and quartz fiber laminate is more powerful than that of the unidirectional carbon fiber laminate; cracks do not occur in the quartz fiber laminate again.

Interestingly, the location of the prefabricated defect will become a weak point as the defect area ratio increases to 5%. When the specimen bore a tensile load, the crack did not only spread from the end of adhesive joint to the interior of carbon fiber laminate but also extended from the prefabricated defect to the edge, quickly causing separation failure between the adhesive layer and laminate, as shown in Figure 6e–h. At this point, the influence of the prefabricated defect on the bonding strength was gradually enhanced, and the failure mode was changed. Comparing the four prefabricated defect types, it can be seen how triangular and square defects not only cause adhesive layer failure but also similarly cause the crack to expand into the interior of the carbon fiber laminate due to the defects’ sharp angle. This phenomenon was more obvious as the angle decreased, further indicating that triangular defects have the most pronounced effect on bonded joints.

As the defect area ratio increased to 10%, owing to the expansion of the prefabricated defect area, the area of the adhesive layer subject to the payload was further reduced, resulting in the disappearance of cracks spreading from the end of bonded joint to the inside of the carbon fiber laminate. Cracks mainly spread from the prefabricated defects to the edges and carbon fiber laminates, causing joint failure (Figure 6i–l). In the meantime, the cracks at the four types of prefabricated defect were significantly higher in number, and the crack bridging was more obvious, subsequently accelerating the energy dissipation rate and leading to a significant decrease in the mechanical properties of the bonded joint.

#### 3.2.2. Effect of Defect Location on Performance

Based on the above analysis, the impact of bonding defect location on the bonding performance for two typical defect shapes, i.e., triangular and circular, at the same area ratio of 5% is shown in Table 3. A schematic of the defect location is shown in the second line of Figure 2a. In contrast to non-defect bonded joints, the bonding strength of triangular defects declined by 19.78%, 18.49%, 12.74% and 9.31%, whereas the bonding strength of circular defects in four positions decreased by 18.00%, 8.51%, 6.98% and 3.86%, respectively. The effect of defect locations on strength reveals significant differences, in which the defects located at the edge have the greatest impact on strength, followed by those at the 0.25 L position and, finally, those at the 0.5 L position. Defects close to the edges of bonded joints were easily coupled with the end of the adhesive layer, forming stress concentration points inducing crack initiation. Comparing the defects at the 0.5 L and 0.5 D positions, cracks extended along the 0.5 L defects in the interface, causing the adhesive layer and the laminate to delaminate; the interface defects have a more serious impact on the performance than the defects within the adhesive layer.

**Figure 7:**
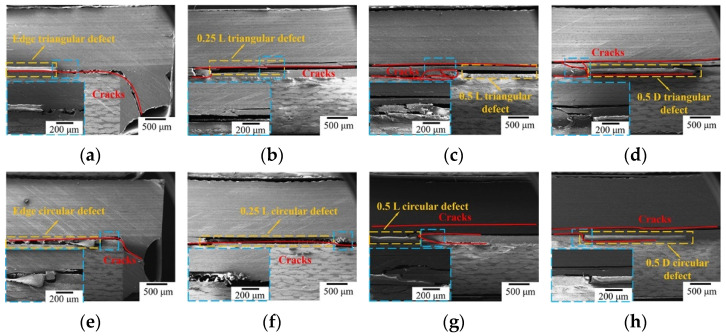
Scanning electron micrographs of samples having defects at different location. (**a**) Triangular defect at edge, (**b**) triangular defect at 0.25 L, (**c**) triangular defect at 0.5 L, (**d**) triangular defect at 0.5 D, (**e**) circular defect at edge, (**f**) circular defect at 0.25 L, (**g**) circular defect at 0.5 L, (**h**) circular defect at 0.5 D.

The crack extensions and failure modes of bonded joints with different positional defects are shown in Figure 7. When the defect is located at the edge of the bonded joint, damage to the joint occurs from the end of the bonded area; the effect of defects is equivalent to reducing the length of the bonding zone, which accelerates the joint failure. Additionally, as can be observed in Figure 7, cracks propagate along the defect to the other end of the interface between the adhesive layer and laminate. As the defect is located at position 0.25 L from the edge, the crack develops from the end of the bonded area and then extends through the defect to the other end, as shown in Figure 7b,f. Compared with the above defect positions, when the defect is located at 0.5 L, the crack is not only generated from the end and expands in the carbon fiber laminate but also spreads from the defect to the end of the adhesive layer and the laminate interior; the overall load-carrying capacity of the specimen is thus improved (Figure 7c,g). When defects are located in the adhesive layer at 0.5 D, cracks are transmitted from within the laminate and expand layer by layer to the defects, as shown in Figure 7d,h. In this time, cracks develop from the interface to the center of adhesive layer defects; then, the adhesive layer is damaged, due to the large amount of energy dissipation, until a large area of adhesive layer bonding failure occurs. Therefore, the degree of weakening of the 0.5 D defect type on joint performance has limitations. According to analysis of the effects of triangular and circular defects in different locations on joint mechanical properties, it has been revealed that, when stress is concentrated at the sharp ends of defects, this tends to cause cracks to propagate outwards. In addition, defects of the tip effect [30,42] are still present; these do not improve with changing defect location. Thus, the mechanical property of components with triangular defects are weaker than those with other types of defects.

#### 3.2.3. Effect of Defect Number on Performance

Circular and triangular defects of four different amounts were, respectively, prefabricated in the bonded area (as shown in the third line of Figure 2a), and the defect area accounted for 5% of the total. Bonding defects with different numbers have demonstrable effects on joint performance, as shown in Table 4. The bonded joint strength with different numbers of triangular defects decreased by 12.74%, 27.01%, 28.90% and 33.44%, and that with circular defects decreased by 6.98%, 11.64%, 13.47% and 21.98%, respectively. Keeping the ratio of the defect area constant, the bonding strength declines as the number of defects increases; the content of triangular defects has a more significant effect on the performance than that of circular defects. Meanwhile, the impact of the defect number on the bonding strength is greater than the defect size. In the case of multiple defects with a certain defect area ratio, cracks are more susceptible to spreading rapidly along the defects, causing the overall failure of the adhesive layer, even though the area size of individual defects has been reduced. As a result, the bond strength can be enhanced by controlling the defect content for a certain defect area ratio.

**Figure 8:**
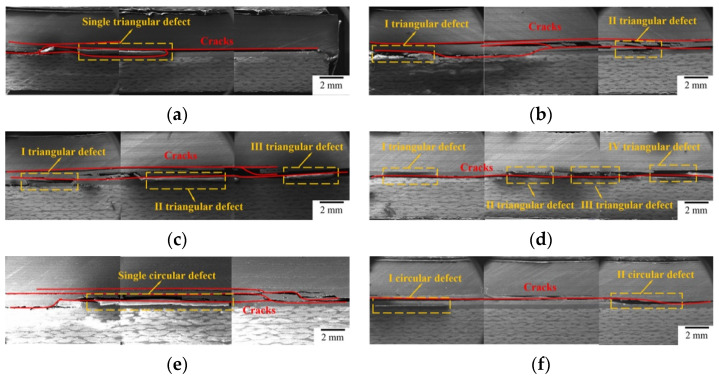

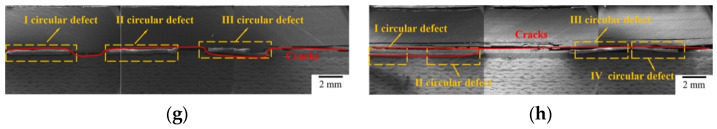
Scanning electron micrographs of samples having defects with different numbers. (**a**) Single triangular defect, (**b**) two triangular defects, (**c**) three triangular defects, (**d**) four triangular defects, (**e**) single circular defect, (**f**) two circular defects, (**g**) three circular defects, (**h**) four circular defects.

In order to directly analyze the mechanism of influence of the defect number on the bonding strength, the failure modes of adhesively bonded joints containing different defect numbers are shown in Figure 8. At a defect number of one, the cracks spread along the laminate, causing failure. The defects in the layer become weak points and cracks grow from the defects to the interface between the adhesive layer and laminate. When the defect number increases to two, the cracks extend from one triangle defect to another until the adhesive layer fails, mainly because these defects easily form stress concentration points, which affects the direction of crack expansion. The crack propagating within the adhesive layer grows outward into the carbon fiber laminate along the circular defects. Similar to a 1% single circular defect, the circular area in the other place cannot change the direction of crack extension due to the smaller monomer area and the further distance between the two defects. As the defect number increases to three or more, the directions of the cracks are directly altered by both triangular and circular defects, which lead to cracks spreading rapidly from one defect to the next. In summary, when the bonded joint is destroyed under load, cracks will propagate along the weak points within the joint. Increasing the defect number increases the density and continuity of defect distribution, providing an effective channel for crack propagation and causing overall failure under low-energy absorption conditions. Furthermore, it can be seen that, under different size, number and location, carbon fiber laminates are always damaged preferentially compared to quartz fiber laminates, but the detailed damage model of the bonded components will change.

#### 3.2.4. Effect of Penetrating Defect on Performance

The relationship between penetration defect and the bonding performance is investigated by prefabricating triangular, rectangular, elliptical and circular defects in the bonded area (as shown in the fourth line of Figure 2a). The defect area ratio is 5% and the defect height is consistent with the thickness of the adhesive film. Compared with that of non-defect bonded joints, the bonding strength of triangular, rectangular, elliptical and circular defects decreases by 42.31%, 30.37%, 18.00% and 8.76%, respectively. Looking at Table 4 for comparison, the joint strength containing triangular, rectangular, elliptical and circular defects decreases by 29.57%, 19.96%, 8.69% and 1.78%, respectively, as shown in Table 5. The penetrating defects have a greater impact on the bonded joint strength than those that are non-penetrating.

There are several inferences to be drawn from these results. Firstly, the continuity of the adhesive layer is broken by a penetrating defect, which reduces the effective load-bearing capacity of the adhesive layer. Secondly, with damage to the layer, the bonded area can not only expand through defects along the interface between the carbon fiber laminate and the adhesive layer but also change the crack direction. Thus, the interface between the quartz fiber laminate and the adhesive layer is destroyed, resulting in the failure and separation of the carbon fiber laminate, the adhesive layer and the quartz fiber laminate. Beyond this, the appearance of geometrical sharp angles in triangular and rectangular defects leads to stress being concentrated in the bonded area; tip-splitting easily forms during energy dissipation, destroying the integrity of the adhesive layer.

**Figure 9:**
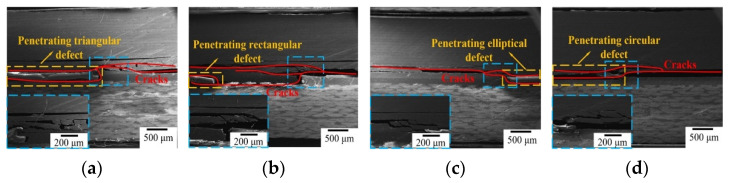
Scanning electron micrographs of samples having penetrating defects with different shapes. (**a**) Triangular defect, (**b**) rectangular defect, (**c**) elliptical defect, (**d**) circular defect.

As shown in Figure 9, continuity in terms of thickness is destroyed by the penetrating defect. The carbon fiber laminate, the prefabricated defects and the quartz fiber laminate are directly connected; then, a closed space in the edge region of the defects is formed by the adhesive film. Once adhesive layer damage occurs, the penetrating defects will directly change the cracks’ direction. This also has a promoting effect on the cracks expanding along the interface, causing the adhesive layer and laminates to peel off. The tip-splitting effect of triangular and rectangular defects is exacerbated by penetrating defects, encouraging cracks to propagate along the end of the defect across the carbon fiber laminate at low energy dissipation. The failure caused by elliptical and circular penetration defects is still mainly controlled by the interfacial separation of the adhesive layer; due to these defects, the crack rapidly results in serious debonding failures in the adhesive layer. By analyzing the mechanical properties and damage modes of joints, it can be inferred that defects of various types within the adhesive layer develop into penetrating defects, thus exacerbating the effect of the defects on the bonding properties of the component and causing the irreversible loss of joint mechanical properties.

### 3.3. Orthogonal Test Analysis of Bonding Process

During the curing process of bonded joints, curing parameters affect joint quality by directly influencing the curing defects in the adhesive layer. The main factors of curing pressure, heating rate, temperature and heat preservation time were selected to perform orthogonal experiments. The results are shown in Table 6.

#### 3.3.1. Difference Analysis

The key factors affecting bonding strength were determined by a difference analysis of the orthogonal tests. As shown in Table 6, the maximum differential value is a heat preservation temperature of 3.82, and the minimum difference value is a heat preservation time of 1.16. Of the four factors, the heat preservation temperature has the greatest effect on the bonding strength, followed by the molding pressure, then the heating rate and, finally, the heat preservation time. Combined with the curing degree analysis, it can be seen that the curing reaction of the adhesive film progresses slowly with a low heat preservation temperature. In a limited process, the three-dimensional mesh structure of the adhesive film is not fully established, causing problems in achieving a strong co-bonding interface with the components to be connected. This result prevents the adhesive film from transmitting and bearing external loads when the components are subjected to loads.

**Figure 10:**
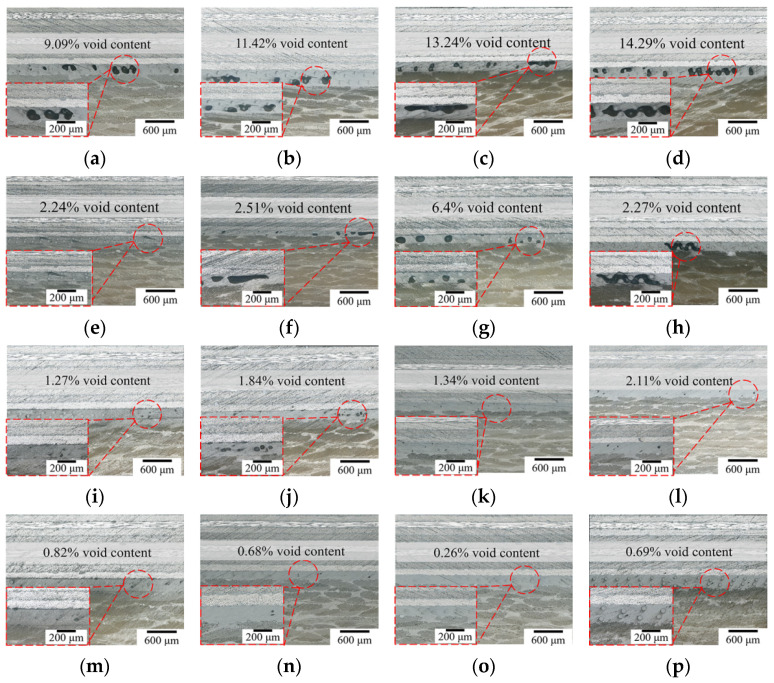
The void morphology and content of bonded region. (**a**–**p**) Orthogonal experiment 1–16.

The TGA test showed that both the adhesive film and the connected component will be thermally decomposed at high heat preservation temperatures, which indicates that thermal decomposition of joint has a weak effect on the adhesive bonding performance when curing temperature is under 200 °C. At heat preservation temperatures more than 160 °C, the adhesive film can complete the curing reaction. Then, the curing pressure will become the main factor and the component performance will be enhanced with it. In the results of the orthogonal experiments shown in Figure 10, five samples were taken from the bonded joints under different curing processes, respectively, and the void area ratios noted in the figure are the average values of the five groups. As can be seen from the micrographs in Figure 10a–d, the void defect area ratio is more than 9% and up to 14.29% at a pressure of 0.0 MPa. Void morphologies evolve into triangular and rectangular forms, and voids defined as penetrating defects are large in number with a random distribution inside the adhesive layer; this weakens the adhesive bonding properties significantly. As shown in Figure 10e–h, as the curing pressure increases to 0.2 MPa, the defect area ratio and number are reduced. There are no longer large rectangular shape defects, but triangular defects with a large impact remain. The adhesive layer is still penetrated by parts of some defects, which limits the adhesive bonding performance. With a further increase in curing pressure, as seen in Figure 10i–l, the void growth is effectively inhibited by a pressure of 0.4 MPa and the area ratio of the void reduces to 2%. Defects are concentrated in the middle of the adhesive layer, and its morphological characteristics are mainly dominated by circles and ellipses. The void area ratio reduces to less than 1% at a pressure of 0.6 MPa, and large voids are crushed under the curing pressure and dissolve again in the resin matrix [43,44], as shown in Figure 10m–p. In this time, small circular voids are only sporadically distributed inside the adhesive layer and are no longer the main factor affecting bonding performance.

Moreover, the dynamic DSC test demonstrated that the exothermic peak of adhesive film was shifted to the right with increased heating rate; following this, both the temperature of the heat preservation platform and the exotherm also increased. This may lead to the uneven distribution of the temperature field in the components, thus affecting the curing cycle of the adhesive layer and weakening its mechanical properties. In particular, the curability analysis showed that the adhesive layer can complete the curing reaction within the corresponding time for all the remaining temperature parameters, except 140 °C. Therefore, heat preservation time has the weakest effect on the performance.

#### 3.3.2. Variance Analysis

We quantitively analyzed the effects of four factors on the tensile strength of the bonded joints, as shown in Table 7.

In the variance results, the *p*-value for the heat preservation time was 0.01, which is lower than the critical value of 0.05 for determining significance. Temperature had a noteworthy effect on the results, followed by curing pressure, while the effects of heating rate and heat preservation time were not significant. The primary reason for this is that, during the bonding process, the film undergoes a phase transition from a solid state to a viscous flow state to a gelatinous state and, finally, to a solid state; the film curing process is directly affected by temperature, ensuring that a full curing cross-linking reaction obviously enhances the bonding strength. In the adhesive film curing process, water vapor and other volatile gases inside the film accumulate to form a gas nucleus, which leads curing pressure to become the main factor affecting void defects within the adhesive layer. A lower curing pressure can cause the voids in the bonding area to continue to grow and become large, randomly distributed triangular and rectangular penetration defects; this has a serious effect on the joint performance [45]. The heating rate and heat preservation time mainly affect the uniformity of the joint temperature field and the curing reaction level. They are not the process parameters that directly affect joint performance, so their relative significant influence level is lower. Overall, a comparison of difference and variance analyses show that the effects of curing parameters on the examined indexes of tensile properties are consistent.

## 4. Conclusions

In this study, we systemically investigated the effects of curing defects in the adhesive layer on the performance of carbon fiber–quartz fiber bonded joints. A differential scanning calorimeter and thermal gravimetric analyzer were used to analyze the isothermal DSC, non-isothermal DSC and TGA of the adhesive film under different temperature conditions. By means of prefabricated defects, the effects of defect morphology, area ratio, content, location and penetration on the bonding performance were studied; the failure forms of different defects were characterized by SEM. Sixteen orthogonal tests were conducted to clarify the influence of heat preservation time, curing pressure, heating rate and heat preservation temperature in terms of the adhesive’s performance. Based on the results, the following conclusions can be drawn:

(1) The J-116B adhesive film releases heat and forms a single exothermic peak after the temperature reaches 160 °C; then, a cross-linking reaction is generated to gradually produce a three-dimensional mesh structure. The exotherm of the adhesive film increases with the heating rate and tends to cause the uneven distribution of the temperature field within the joints. The required curing time is the shortest at a heating preservation temperature of 180 °C, which is conducive to optimizing and adjusting the curing process.

(2) The adhesive film and resin matrix enter the first stage of thermal degradation after the temperature reaches 200 °C; the heat losses due to volatilization of small organic molecules are 0.30% and 0.58%, respectively. The volatile heat losses of organic macromolecules can reach 69.98% and 66.32% when the temperature exceeds 300 °C. These results indicate that, once the heat loss stage is entered, the molecular structure of the adhesive layer and laminate will be destroyed and the load-bearing properties of the joints will be seriously affected.

(3) For single-shape defects, an increase in area ratio can weaken the adhesive’s performance. A defect area ratio of 5% can directly affect crack initiation and propagation; a larger defect area ratio can enhance the sensitivity of the bonding strength to the defect shape. In contrast, with the same area ratio, triangle defects have the most significant impact on the performance due to the tip-splitting effect.

(4) Defects near the edge of the bonded area with a certain area ratio more easily couple with the end of the adhesive layer to produce a stress concentration point. The defects inside the adhesive layer (0.5 D) need to absorb more energy in order for the adhesive layer to be destroyed, and their influence on the bonding performance is limited. As the defect content increases, the continuity of the adhesive layer in the length direction is disrupted, thus making it easier for cracks to propagate rapidly along the defects and resulting in the interfacial debonding failure of the adhesive layer.

(5) Penetration defects have the most serious impact on the joint performance by breaking the continuity of the adhesive film in the thickness direction and changing the direction of cracks; this promotes crack expansion along the bonded layer interface.

(6) The heat preservation temperature significantly affects the mechanical properties of joints, mainly by interfering with the curing process. After the adhesive film successfully completes the curing reaction, the void morphology and distribution inside the adhesive layer are affected by the curing pressure, thus changing the joint performance. The heating rate and the heat preservation time exert a less significant influence.

## Figures and Tables

**Table 1:** Factors and levels of the orthogonal experiment.

	Factors	Curing Pressure (MPa)	Heating Rate (°C/min)	Heat Preservation Temperature (°C)	Heat Preservation Time (min)
Levels					
1		0.0	0.5	140	120
2		0.2	1	160	150
3		0.4	3	180	180
4		0.6	5	200	210

**Table 2:** Experimental results of defect samples with different shapes and area ratios.

Defect Shape	Area Ratio of Defect (%)	Tensile Shear Strength (MPa)	Relative Tensile Shear Strength (%)
NA	NA	16.33	100
Circular	1	15.71	96.20
Elliptical	1	15.57	95.35
Rectangular	1	15.22	93.20
Triangular	1	14.96	91.61
Circular	5	15.19	93.02
Elliptical	5	14.81	90.69
Rectangular	5	14.63	89.59
Triangular	5	14.25	87.26
Circular	10	15.16	92.84
Elliptical	10	14.69	89.96
Rectangular	10	14.32	87.69
Triangular	10	14.13	86.53

**Table 3:** Experimental results of samples having defects at different locations.

Defect Shape	Area Ratio of Defect (%)	Defect Location	Tensile Shear Strength (MPa)	Relative Tensile Shear Strength (%)
NA	NA	NA	16.33	100
Circular	5	Edge	13.39	82.00
Circular	5	0.25 L	14.94	91.49
Circular	5	0.5 L	15.19	93.02
Circular	5	0.5 D	15.70	96.14
Triangular	5	Edge	13.10	80.22
Triangular	5	0.25 L	13.31	81.51
Triangular	5	0.5 L	14.25	87.26
Triangular	5	0.5 D	14.81	90.69

**Table 4:** Experimental results of samples having defects with different numbers.

Defect Shape	Area Ratio of Defect in Total (%)	Defect Number	Tensile Shear Strength (MPa)	Relative Tensile Shear Strength (%)
NA	NA	NA	16.33	100
Circular	5	1	15.19	93.02
Circular	5	2	14.43	88.36
Circular	5	3	14.16	86.53
Circular	5	4	12.74	78.02
Triangular	5	1	14.25	87.26
Triangular	5	2	11.92	72.99
Triangular	5	3	11.61	71.10
Triangular	5	4	10.87	66.56

**Table 5:** Experimental results of samples having penetrating defects.

Defect Shape	Area Ratio of Defect in Total (%)	Penetrating or Non-Penetrating	Tensile Shear Strength (MPa)	Relative Tensile Shear Strength (%)
NA	NA	NA	16.33	100
Circular	5	penetrating	14.90	91.24
Elliptical	5	penetrating	13.39	82.00
Rectangular	5	penetrating	11.37	69.63
Triangular	5	penetrating	9.42	57.69

**Table 6:** Tested results of tensile shear strength in orthogonal experiment.

Test Number	Curing Pressure (MPa)	Heating Rate (°C/min)	Heat Preservation Temperature (°C)	Heat Preservation Time (min)	Tensile Shear Strength (MPa)
1	1	1	1	1	12.65
2	1	2	2	2	15.65
3	1	3	3	3	15.25
4	1	4	4	4	10.62
5	2	1	2	3	16.39
6	2	2	1	4	16.45
7	2	3	4	1	11.15
8	2	4	3	2	15.45
9	3	1	3	4	16.61
10	3	2	4	3	13.98
11	3	3	1	2	14.25
12	3	4	2	1	15.89
13	4	1	4	2	14.22
14	4	2	3	1	17.62
15	4	3	2	4	17.34
16	4	4	1	3	16.32
Mean value I	13.54	14.97	14.92	14.33	
Mean value II	14.86	15.92	16.32	14.89	
Mean value III	15.18	14.50	16.23	15.49	
Mean value IV	16.37	14.57	12.49	15.25	
Rang R	2.83	1.35	3.82	1.16	

**Table 7:** Variance analysis of cure pressure, heating rate, cure temperature and heat preservation.

Factor	Deviation Sum of Squares	Degree	Mean Square	F Value	Salience
Curing pressure	16.270	3	5.423	13.19	0.031
Heating rate	5.175	3	1.725	4.20	0.135
Heat preservation temperature	38.195	3	12.732	30.97	0.009
Heat preservation time	3.055	3	1.018	2.48	0.238
Error	1.233	3	0.411		

## Data Availability

The data presented in this study are available on request from the corresponding author due to privacy.
